# Supplementary material for: Empirical methods for controlling false positives and estimating confidence in ChIP-Seq peaks
Source: BMC Bioinformatics. 2008 Dec 5;9:523. doi: 10.1186/1471-2105-9-523 (PMC2628906; doi:10.1186/1471-2105-9-523)
Supplement: Additional File 1 — A variety of html documents from the USeq web site detailing the available applications, their best usage, output file type descriptions, command line menus, etc. [file 1471-2105-9-523-S1.zip › USeqUserGuides/JavaDoc.zip!/JavaDoc/util/bio/annotation/class-use/Translation.html]

Uses of Class util.bio.annotation.Translation


|  |  |  |
| --- | --- | --- |
| **Overview** \| **Package** \| **Class** \| **Use** \| **Tree** \| **Deprecated** \| **Index** \| **Help** | |  |
| PREV   NEXT | **FRAMES**    **NO FRAMES**     **All Classes** |  |


---


## **Uses of Class util.bio.annotation.Translation**

| Packages that use Translation | |
| --- | --- |
| **util.bio.annotation** | For representing gene annotation. |

Uses of Translation in util.bio.annotation

| Methods in util.bio.annotation that return Translation | |
| --- | --- |
| `Translation` | `TransGroup.getTranslation()` |

Constructors in util.bio.annotation with parameters of type Translation

`TransGroup(Transcript transcript,
Translation translation,
ExonIntron[] exons)`

---


|  |  |  |
| --- | --- | --- |
| **Overview** \| **Package** \| **Class** \| **Use** \| **Tree** \| **Deprecated** \| **Index** \| **Help** | |  |
| PREV   NEXT | **FRAMES**    **NO FRAMES**     **All Classes** |  |


---
